# Percutaneous microwave coagulation for eradication of VX2 tumors subcutaneously in rabbits

**DOI:** 10.1186/1477-7819-10-97

**Published:** 2012-05-30

**Authors:** Wenbin Zhou, Qiang Ding, Xiaoan Liu, Yanni Jiang, Ling Chen, Yifen Zhang, Tiansong Xia, Shui Wang

**Affiliations:** 1 Department of Breast Surgery, The First Affiliated Hospital with Nanjing Medical University, 300 Guangzhou Road, Nanjing, 210029, China; 2 Department of Radiology, The First Affiliated Hospital with Nanjing Medical University, 300 Guangzhou Road, Nanjing, 210029, China; 3 Department of Pathology, Nanjing Drum Tower Hospital, Nanjing University Medical School, 321 Zhongshan Road, Nanjing, 210008, China

**Keywords:** Percutaneous microwave coagulation, Output power, VX2, Blood-flow

## Abstract

**Background:**

Percutaneous microwave coagulation (PMC) has been accepted as a promising modality in the treatment of tumors in well-vascularized tissues such as liver tumors and hysteromyoma. However, PMC for treatment of tumors in low blood-flow tissues has been seldom reported. The aim of this study was to determine the feasibility and safety of PMC for the treatment of tumors in low blood-flow tissues in a rabbit model.

**Methods:**

Fifteen rabbits with VX2 tumors implanted subcutaneously underneath the right second nipple were divided into a PMC group (n = 9) and a control group (n = 6). PMC was performed with output power of 40 W for one to two minutes. The therapeutic efficacy was evaluated by magnetic resonance imaging (MRI), physical examinations, survival rate, and histology. The cosmetic outcome after PMC was also assessed.

**Results:**

In the PMC group, tumor eradication was achieved in six rabbits (66.7%) without any evidence of tumor recurrence and metastasis as proven by MRI and histological examinations. The mean greatest and shortest tumor diameters of these six rabbits were 1.83 and 1.33 cm, respectively. Slight epidermal burns, which proved reversible, were found in seven rabbits (77.8%). The PMC group had a significantly longer survival than those in the control group (*P* = 0.0097). The four rabbits with coagulated tumors survived more than three months with their tumors becoming nonpalpable and undetectable by MRI and histological examinations.

**Conclusions:**

PMC is feasible and safe in the treatment of tumors in low blood-flow tissues in a rabbit model. Attention should be paid to avoid skin burns with PMC.

## Background

Minimally invasive therapies, including radiofrequency ablation (RFA), cryotherapy, high-intensity focused ultrasound, and laser therapy, have been widely used in the treatment of solitary tumors [1-5]. Due to the advantages of favorable efficacy, low complication rates and optional use of multiple antennae, RFA is the currently widely used thermal ablation technique [6,7]. Percutaneous microwave coagulation (PMC), a new promising modality, has been used in the treatment of hepatic tumors as an effective therapy [8-10]. Compared to other minimally invasive therapies, PMC is simpler to operate and has a higher potential for complete tumor ablation with many advantages [8,11,12], such as improved convection profile, consistent higher intratumoral temperature, larger ablation volume, and shorter ablation time. Previous studies [8,12,13] reported that a large volume (maximum size >3cm) can be ablated by PMC *in vivo* with high power outputs (≥60 W at 2,450 MHz). In addition, the temperature at the site 5 mm away from the electrode was higher with microwave compared with RFA [12]. Therefore, PMC may elevate the temperature more quickly in a shorter time for complete tumor ablation.

The blood-flow is known to affect the efficacy of minimally invasive therapies. Theoretically, a larger volume of ablation can be achieved in low blood-flow tissues relative to high blood-flow tissues. In fact, high output power (60–80 W) has been widely used in the treatment of tumors in high blood-flow tissues [8,14-16]. For example, PMC typically used high (60–80 W) power outputs for the treatment of tumors in well-vascularized tissues such as liver tumors and hysteromyoma [9,13,16]. However, PMC in the treatment of tumors in low blood-flow tissues has not been reported. Obviously, PMC using high (60–80 W) power outputs in low blood-flow tissues may cause more serious injuries to surrounding tissues. Moreover, low blood-flow can affect the evolution of the ablated tumors. For example, the palpable ablated mass in the breast after minimally invasive therapies for a long period of time will cause discomfort and anxiety to patients [17,18]. Detailed information about ablated tumors in low blood-flow tissues, including the breast, is not very clear.

Other minimally invasive therapies [19-22] have been attempted to ablate tumors in low blood-flow tissue including the breast. Previous study [23] showed the usefulness of microwave coagulation to breast cancer *ex vivo*. The VX2 tumor model is a widely used animal tumor model for minimally invasive therapies [24-29]. We implanted the VX2 tumors subcutaneously underneath the right second nipple in rabbits to investigate whether a relatively low output power 60 W at 2,450 MHz was suitable for ablating the tumors in low blood-flow tissues. However, serious burns to the skin and muscles were observed in our preliminary experiment. The aim of this study was to determine the feasibility and safety of PMC with a lower output power (40 W at 2,450 MHz) for the treatment of tumors in low-blood flow tissues. The evolution of PMC ablated tumors in low-blood flow tissues was also assessed.

## Methods

### Tumor model

Animal experiments were approved by the Animal Care and Use Subcommittee at our University. Adult female New Zealand White rabbits weighing between 2.0 and 3.0 kg were used in this study. The VX2 cell line, used for tumor implantation, was obtained from the Surgery Department of our Hospital. The rabbits were anesthetized using an intravenous injection of pentobarbital (30 mg/mL, 0.8 to 1.2 mL/kg, pentobarbital sodium; Sigma, St, Louis, MO, USA) before the VX2 tumor inoculation and other procedures. The required tumor tissue, taken from a tumor carrier rabbit, was cut into small strips (1.5 × 1.5 × 6 mm). Each strip of tumor tissue was inserted subcutaneously underneath the right second nipple by a 16-gauge needle. The animals were followed up every week by physical examination. The tumor growth was 100% in the implantation sites. When the animals refused solid and fluid intake for more than four days with concomitant apathy and weight loss of more than 20%, the animals were sacrificed for ethical reasons [29].

### Experimental groups

The larger and shorter diameters of the tumor were measured using a caliper. After the larger diameter of the tumor reached about 15 mm (10 to 21 days), 15 rabbits were included in this study with the following treatments: PMC group (n = 9), nine rabbits with nine VX2 tumors were treated with PMC and control group (n = 6), six rabbits with six VX2 tumors served as untreated controls.

### PMC protocol

After the same anesthesia as in tumor implantation, the rabbits were positioned supine. The cooled-shaft antenna (2 mm in diameter) was inserted into the tumor along the long axis without any navigation. This microwave delivery system consists of a microwave generator, a flexible coaxial cable and an internally-water-cooled-shaft antenna. The microwave irradiation frequency is 2,450 MHz. An output power of 40 W was chosen in this study. After testing the cold water (4°C) cycling system, the PMC procedure was started for one to two minutes to eradication of the entire tumor according to the tumor size [8,11-13] and our own experience. Since the larger diameter of all the tumors was less than 2.5 cm, two minutes may be enough. Therefore, when the shorter diameter of the tumor was shorter than 1.5 cm, one minute was chosen. When the shorter diameter of the tumor was shorter than 2.0 cm, one and a half minutes was used. When the shorter diameter of the tumor was shorter than 2.5 cm, two minutes was chosen in our study.

After PMC treatment, the antenna was removed and the incision closed. All the rabbits were monitored during the PMC treatment and for one month after surgery for any complications that might be induced by the intervention. The degree and extension of the burns to the skin were evaluated. The behavior of the rabbits after the treatment was observed. Furthermore, the weight and food intake of the rabbits were monitored.

### Therapeutic evaluation

The efficacy of PMC therapy was evaluated with survival rate, MRI, physical examination of the coagulated tumors and histopathology. MRI was performed to monitor the long-term results of the coagulated tumors at day 0, week 1, week 3 and week 7 after PMC therapy. Core needle biopsy was randomly performed on two rabbits for histological examination at week 1 and week 3 after PMC therapy. Since there was little tissue under the skin at week 7, core needle biopsy was not performed at this time-point. Incomplete ablation was determined by core needle biopsy or MRI follow-up without treatment control. When the tumors were not reabsorbed with local growth or the needle biopsies were positive, the tumors were recognized as incomplete ablation. Survival rate was used to assess the therapeutic effect between the two groups.

### MRI follow-up

Prior to MRI scanning, the animals were anesthetized by the procedure described as in tumor implantation to decrease respiratory rates for clear images. The animals were strapped but not intubated to reduce motion artifacts. Respiratory-triggered sequence with prospective acquisition correction was applied to MRI examination for respiration control. Rabbits in the PMC group were subject to three Tesla (T) MRI examinations (Trio, Siemens Healthcare) at pre-microwave therapy, one week, three weeks and seven weeks post-PMC therapy. T2-weighted 2D fast spin-echo (FSE) sequences were acquired in the axial plane using a phased-array coil. The 2D FSE sequence was performed with the following parameters: TR, 3,948 to 4,960 milliseconds; TE, 80 to 89 milliseconds; matrix size (168 to 256) × 320; field of view, (135 to 150) × (150 to 180) mm; slice thickness, 4 mm; bandwidth, 205 to 260 Hz; and acquisition time, 104 to 150 seconds. The techniques of MRI were the same during the procedure.

### Histopathological examination

The animals were autopsied within six hours after death. The tissue specimens were fixed in 10% formalin solution, embedded in paraffin, sectioned into 4 μm slices, and stained with H & E. The histological slides were evaluated by one experienced pathologist.

### Statistics

The numerical data were reported as mean ± standard deviation (SD). Survival curves were generated using the Kaplan-Meier method. The log-rank test was used to compare mean survival between the two groups. All *P*-values were two-tailed with 5% significance levels. All statistical analyses were performed using STATA version 11.0 (Computer Resource Center, America).

## Results

### General aspects

All rabbits in the PMC and control groups tolerated the experimental procedures. All the animals recovered well after the experiment. Although output power was only 40 W, epidermal burns, measuring 0.5 to approximately 1 cm in the greatest dimension, were found in seven rabbits in the PMC group. However, no necrosis of the skin was observed. Due to a high rate (7/9, 77.8%) of epidermal burns, it seems a very likely occurrence. No other immediate adverse effects occurred in all rabbits. Only about ten minutes was needed for the total PMC procedure.

### Tumor eradication effect

**Figure 1:**
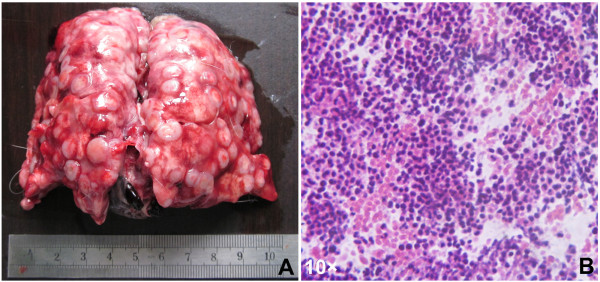
**One case with lung metastases. A**) Macroscopic appearances of the lungs with metastatic nodules; **B**) Metastatic nodules demonstrated by hematoxylin and eosin (H&E) stain (10 ×).

**Figure 2:**
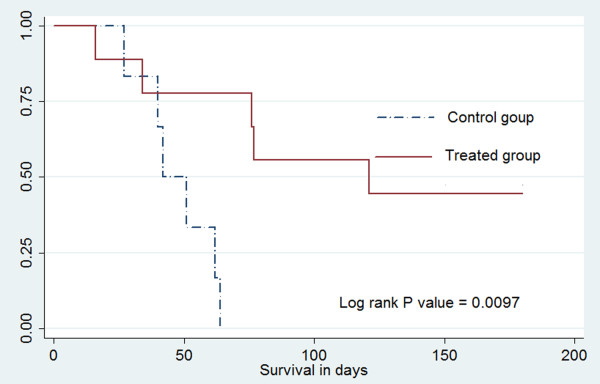
**Graph of Kaplan-Meier survival analysis showed that rabbits in the PMC-treated group had a significantly higher survival than those in the control group**.

The detailed information is shown in Table 1. The rabbits in the control group without PMC therapy died of end-stage malignancies (mean 47.7 ± 14.2 days). Five rabbits in the control group showed pulmonary metastases (Figure 1). In the PMC group, the mean greatest diameter of the tumor was 1.72 ± 0.36 cm (range, 1.5 to 2.5 cm) before PMC therapy. The mean coagulation time was 1.06 ± 0.17 minutes (range, 1.0 to 1.5 minutes). Tumor eradication was achieved in six rabbits (66.7%) without any evidence of tumor recurrence and metastasis. The mean greatest tumor diameter of these six rabbits was 1.83 ± 0.41 cm (range, 1.5 to 2.5 cm), and the short diameter was 1.33 ± 0.41 cm (range, 1.0 to 2.0 cm) before PMC treatment. Of these six rabbits, two rabbits died on the 16th and 34th day after PMC therapy without evidence of tumor recurrence or metastasis. An infectious disease, which may be caused by the PMC therapy, induced the death on day 16 after PMC treatment. Diarrhea, which is one of the most common causes of death in immature rabbits, induced the death on day 34 after PMC therapy. The other four rabbits survived longer than three months free of disease, of which three rabbits survived longer than six months. In contrast, no rabbits in the control group survived longer than 70 days. Survival analysis showed that rabbits in the PMC group had a significantly higher survival than those in control group (*P* = 0.0097) (Figure 2).

**Table 1:** Summary of PMC tumor ablation effects in the breast

**Group**	**No. animals**	**Local growth**	**Metastasis**	**Eradication**	**Eradication rate (%)**	**Survive >3 months**
PMC	9	3	2	6	66.7	4
Control	6	6	6	0	0	0

### Physical examination and MRI findings

Tumor palpability was evaluated by physical examination every week. One week after PMC, the size of the ablated area was larger than before. Except for three rabbits with local recurrence, the tumors treated with PMC in the other six rabbits were progressively absorbed. Of these six rabbits, four survived longer than three months. All the tumors of these four rabbits became nonpalpable in three months after PMC therapy. The epidermal burns in the rabbits were healed after approximately 2 to 3 weeks.

**Figure 3:**
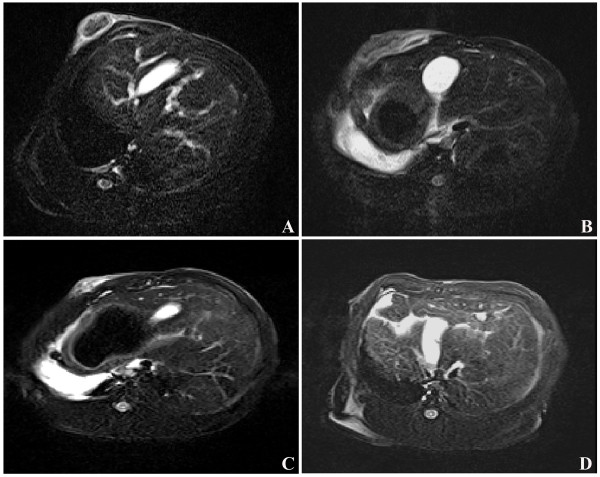
**Magnetic resonance (MR) images from a rabbit with a VX2 tumor before and after PMC therapy. A**) Image obtained before microwave therapy; **B**) Image obtained one week after PMC therapy: edema and hyperemia of lesion is obvious; **C**) Image obtained three weeks after microwave therapy: most part of the ablated tumor was absorbed; **D**) Image obtained seven weeks after microwave therapy: the ablated tumor was absorbed completely to nonpalpable.

Progressive resorption was also detected by MRI. The details are shown in Figure 3. In accordance with physical examinations, the image at one week (Figure 3B) after PMC therapy showed obvious edema and hyperemia around the coagulated tumor. At week three after PMC therapy (Figure 3C), the edema and hyperemia of the lesion was partly absorbed and the size of the coagulated area was much smaller than that at week one. The coagulated VX2 tumor was absorbed completely at week seven after PMC therapy (Figure 3D).

**Figure 4:**
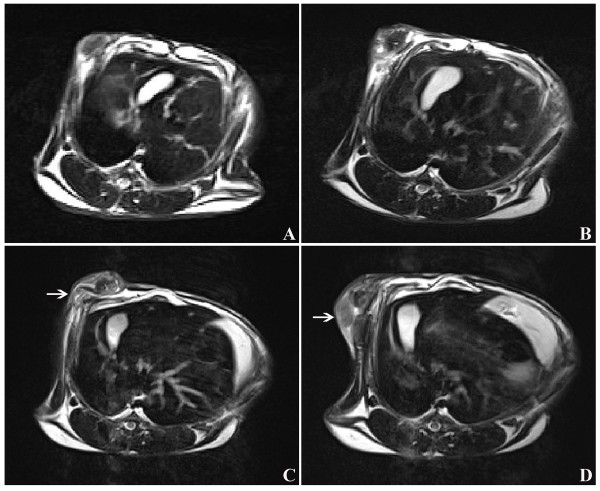
**Magnetic resonance (MR) images from a rabbit with local recurrence after PMC therapy. A**) Image obtained before microwave therapy: a tumor beneath the skin; **B**) Image obtained one week after PMC therapy: obvious edema and hyperemia around the ablated tumor was observed; **C**) Image obtained three weeks after microwave therapy: the ablated tumor was partly absorbed; however, local recurrence (arrow) was detected although it was not very clear; **D**) Image obtained seven weeks after microwave therapy: most of the ablated tumor was absorbed; however, obvious local recurrence (arrow) was detected.

Local recurrence was detected in three rabbits in the PMC group by MRI (Figure 4). At one week after PMC therapy, no obvious local recurrence was detected by physical examination and MRI (Figure 4B) due to obvious edema and hyperemia. However, local recurrence was detected by MRI at week three after PMC therapy, but it was not very clear (Figure 4C). At week seven after PMC therapy, local recurrence was very obvious (Figure 4D). Of these three rabbits with local recurrence, metastases were observed in two rabbits. The first metastasis was detected by MRI 35 days after PMC therapy.

### Histopathological examination

**Figure 5:**
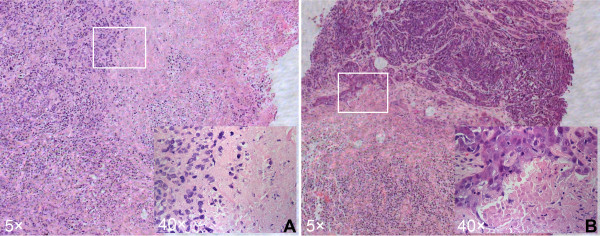
**Photomicrographs from coagulated VX2 tumor and viable tumor stained with H & E at week one (A) and week three (B)**.

Histologically, the coagulated tissues appeared almost unchanged after PMC therapy, especially at week one (Figure 5A). At the H & E staining, the coagulated tumors looked almost intact in tissue architecture and were stained a little lighter than viable tissues. The outline of the coagulated tumor cells was still clear, but the nuclei were fractured. Some apoptotic bodies were observed in the area, the coagulation of which was insufficient. The cell architecture in the viable area was clearer at week three than week one. The difference between the coagulated tumors and viable tissues was more obvious at week three than week one (Figure 5). A sharper demarcation between the coagulated tumors and viable tissues was observed at week three after PMC therapy than at week one.

## Discussion

The present study first reported a successful experience of PMC with a low output power (40 W at 2450 MHz) in the treatment of VX2 tumors in low blood-flow tissues. Our results suggest that PMC with a low output power (40 W) is feasible in the treatment of tumors in low blood-flow tissues. However, slight epidermal burns without necrosis of the skin were still found. Furthermore, the ablated tumors were well absorbed to nonpalpable in low blood-flow tissues.

RFA has been widely used in the treatment of different tumors in VX2 tumor models [25-27,29]. Previous studies suggested rabbits in RFA-treated groups survived longer than those in control groups. We first reported PMC in the treatment of tumor implantations subcutaneously underneath the right second nipple in rabbits, one kind of low blood-flow tissues. Although the VX2 tumor cell line is known for its malignant potential and ability to metastasize early, the same encouraging results were still observed in this study. In the PMC group, three of nine rabbits with local recurrence showed incomplete ablation, determined by core needle biopsy or MRI follow-up assessment without treatment control. However, six other rabbits, that survived for a long period, showed complete ablation. Future studies should be carried out to confirm this interesting finding in clinic.

Although the output power was low in this study, skin burns were still observed in seven rabbits in the PMC group. The burns were caused by a non controlled exposure that heated surrounding tissues that had a low flow and, therefore, burned easily. Therefore, burns to the tissues around the tumor should be considered when PMC is used, especially for tumors in low blood-flow tissues. For example, to avoid burns in the skin or pectoralis major muscle, the distance from the breast tumor to the skin and pectoralis major muscle should be considered. According to previous studies [30,31], a distance of at least 1 cm between the tumor and the skin and between the tumor and the chest wall is required. Whether this criterion is suitable for PMC in the treatment of small breast cancers should be confirmed. In addition, a previous study [32] suggested that the extension of microwave exposure could be controlled by using MR thermometry. The microwave generator at 2,450 MHz did not interfere with MR images. Therefore, MR thermometry can be used to control the temperature at the target level for PMC therapy. However, the VX2 tumors were easy to target and MR was not used for navigation in this study. For clinical accuracy and safety, future studies should be undertaken to determine the role of MR thermometry in PMC therapy.

PMC has gradually been accepted as a promising new modality for tumor ablation, with many advantages. In our study, only about one minute was used for complete ablation. The treatment time was far less than other minimally invasive therapies [28,33]. In addition, PMC was simpler to operate compared with other minimally invasive therapies. Only about ten minutes was needed in total for the therapy procedures in our study. Our results suggest that PMC with a low output power may be a promising modality for the treatment of tumors in low blood-flow tissues.

Although our results are encouraging, several concerns should be considered for clinical practice of PMC in the treatment of tumors in low blood-flow tissues including breast cancers. First, for complete ablation with a relatively low output power, tumors of a large size should be excluded. Second, the guidance of the antenna is important for accurate placement. Third, the monitoring of the procedure is also important. Future clinical studies are needed to confirm our encouraging results.

Several limitations still existed in this study. First, the VX2 tumors were present beneath the skin, which enabled us to accurately target the tumor and control the lesion size. Second, since no rabbit-specific coils or scanner was used for the MRI scanning, the images were not very clear. Not using any technique to control complete ablation right after the treatment was an important limitation to the technique since the potential for relapse is high and can only be ascertained after the relapse occurs. Contrast enhanced imaging should be used to determine whether the whole tumor is ablated in the future. Third, although rabbits in the PMC group survived significantly longer than those in the control group, future studies with a large sample size are still needed to confirm our encouraging results.

## Conclusions

The results of this study have demonstrated the feasibility of PMC in treating VX2 tumors in low blood-flow tissues. However, slight epidermal burns without necrosis of the skin, which proved reversible, were still found. PMC was effective and safe in the treatment of tumors in low blood-flow tissues. After microwave coagulation, the tumors were well absorbed to nonpalpable in low blood-flow tissues. Future clinical studies are required to validate the use of this technique in the treatment of tumors in low blood-flow tissues including breast cancers and metastatic tumors subcutaneously.

## Abbreviations

FSE, fast spin-echo; H & E, hematoxylin and eosin; MRI, magnetic resonance imaging; PMC, percutaneous microwave coagulation; RFA, radiofrequency ablation; T, tesla.

## Competing interests

The authors declare that they have no competing interests.

## Authors’ contributions

SW has contributed to the conception and design of the study, the analysis and interpretation of data, the revision of the article as well as final approval of the version to be submitted. WZ, QD and XL participated in the design of the study, performed the statistical analysis, drafted and revised the article. WZ, LC, and TX performed the experimental study; YJ performed the MRI. YZ performed the histopathological examinations. All authors read and approved the final version of the manuscript.
